# Risk of Cancer in a Community Exposed to Per- and Poly-Fluoroalkyl Substances

**DOI:** 10.1177/11786302221076707

**Published:** 2022-02-11

**Authors:** Mindi F Messmer, Jeffrey Salloway, Nawar Shara, Ben Locwin, Megan W Harvey, Nora Traviss

**Affiliations:** 1 VistaNova Consulting, Rye, NH, USA; 2 NH Science and Public Health, Portsmouth, NH, USA; 3 Department of Health Management and Policy, University of New Hampshire, Durham, NH, USA; 4 Georgetown-Howard Universities Center for Clinical and Translational Science, Washington, DC, USA; 5 MedStar Health Research Institute, Washington, DC, USA; 6 Private Consultant, Portsmouth, NH, USA; 7 Springfield College, School of Health Sciences, Springfield, MA, USA; 8 Environmental Studies, Keene State College, Keene, NH, USA

**Keywords:** PFAS, PFOA, drinking water, air emissions, cancer

## Abstract

**Background:**

Per- and polyfluoroalkyl substances (PFAS) emissions from a plastic coating industrial source in southern New Hampshire (NH) have contaminated at least 65 square miles of drinking water. Prior research indicates that high levels of PFAS are associated with a variety of adverse health outcomes, including an increased risk of cancer. Reports indicate that mean blood serum levels of perfluorooctanoic acid (PFOA), one type of PFAS, in residents of the exposed community are more than 2 times greater than the mean blood serum level in the US. Merrimack public water supply customers also have higher average blood levels of perfluorooctane sulfonic acid (PFOS) and perfluorohexane sulfonic acid (PFHxS) than the time—matched US average. A 2018 report concludes that the incidence rate of cancer in Merrimack does not exceed the incidence rate of cancer in NH in general. However, prior reporting on the risk of cancer in Merrimack is compared only to a state-wide metric influenced by the Merrimack cancer incidence.

**Methods:**

Our ecological study compared the risk in Merrimack, NH residents for 24 types of cancer between 2005 and 2014, targeted in a previous study, and all-cause cancers, to US national cancer rates and cancer rates in demographically similar towns in New England. Four New England “unexposed towns” were chosen based on demographic similarity to Merrimack, with no documented PFAS exposure in water supplies. We utilized unadjusted logistical regression to approximate risk ratios (RR) and 95% confidence intervals (CI) assessing the risk of cancer in Merrimack NH to each of the 4 comparator communities, the pooled comparator variable, and national average incidence.

**Results:**

Residents of Merrimack, NH experienced a significantly higher risk of thyroid cancer (RR = 1.47, 95% CI 1.12-1.93), bladder cancer (RR = 1.45, 95% CI 1.17-1.81), esophageal cancer (RR = 1.71, 95% CI 1.1-2.65), and mesothelioma (RR = 2.41, 95% CI 1.09-5.34), compared to national averages. Our work also suggests that Merrimack residents experienced a significantly higher risk of all-cause cancer (RR = 1.34, 95% CI 1.25-1.43), thyroid cancer (RR = 1.69, 95% CI 1.19-2.39), colon cancer (RR = 1.27, 95% CI 1.02-1.57), and prostate cancer (RR = 1.36, 95% CI 1.15, 1.6) compared with similarly exposed New England communities. Our results indicate that residents of Merrimack may also have a significantly lower risk of some site-specific cancers compared to national averages, including lower risk of prostate cancer (RR = 0.57, 95% CI 0.5-0.66), female breast cancer (RR = 0.60, 95% CI 0.52-0.68), ovarian cancer (RR = 0.52, 95% CI 0.33-0.84) and cervical cancer (RR = 0.29, 95% CI 0.12-0.69).

**Conclusion:**

Merrimack residents experienced a significantly higher risk of at least 4 types of cancer over 10 years between 2005 and 2014. Merrimack is a community with documented PFAS contamination of drinking water in public and private water sources. Results indicate that further research is warranted to elucidate if southern NH residents experience increased risk for various types of cancer due to exposure to PFAS contamination.

## Background

In March of 2016, perfluorooctanoic acid (PFOA) was detected in the Merrimack, NH public drinking water supply at concentrations above the US Environmental Protection Agency (USEPA) lifetime health advisory of 70 parts per trillion (ppt). The NH Department of Environmental Services (DES) subsequently ordered the shutdown of 2 public water supply wells.^1^ No PFAS mitigation efforts were undertaken for public or private water supplies before or between 2005 and 2014.

**Figure 1.**
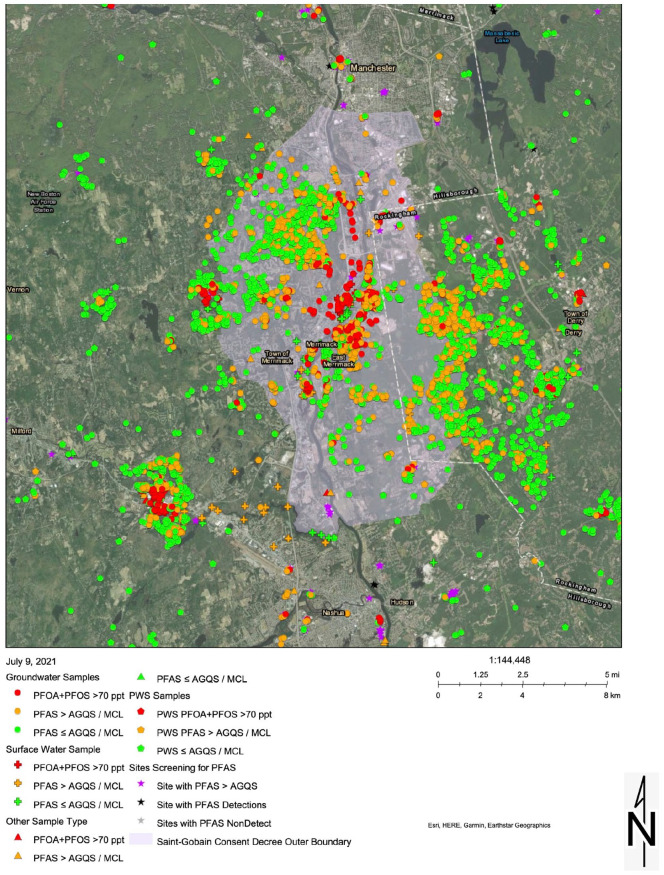
PFAS water results, southern NH investigation. Source: Retrieved from https://nhdes.maps.arcgis.com/.

PFOA contamination in the public drinking water in Merrimack, Bedford, Londonderry, Manchester, and Litchfield was traced to emissions from Saint Gobain Performance Plastics (Saint Gobain) located in Merrimack, NH. In total, 65 square miles encompassing portions of approximately these 5 towns (Figure 1) experienced ground-water contamination of PFOA.^2^ The full extent of the contamination is still under investigation because stricter drinking water standards were imposed for 4 PFAS chemicals (PFOA, perfluorooctane sulfonic acid [PFOS], perfluorohexane sulfonic acid [PFHxS], and perfluorononanoic acid [PFNA]) in July 2020.^3^

As part of the investigation, the USEPA collected air samples from stack emissions at the Saint Gobain plant in 2019. USEPA identified 190 PFAS substances in the samples, 101 of which are novel.^4^ As of 2021, a regenerative thermal oxidizer was reportedly installed to address PFAS emissions at the Saint Gobain plant.

In 2000, Saint Gobain acquired ChemFab, which operated at the Merrimack plant since the 1980s, and moved to Merrimack from Bennington, Vermont (VT). Saint Gobain produces polytetrafluoroethylene (PTFE)-coated glass and other fabrics, sports dome roofs, radomes, and other defense industry products. According to Saint Gobain, operations at the Merrimack plant include fluoropolymer coating application to glass cloth, where fabric sheets are dried and cured at high temperatures with venting through stacks on the factory’s roof.^5^ Saint Gobain still produces ChemFab fabrics and manufactures Vetrotex, an industrial fabric made from glass fibers coated in polytetrafluoroethylene (PTFE), the compound used in Teflon™.^6^

PFAS are persistent in the environment and bioaccumulate in humans, animals, and fish. Human exposure to PFAS is widespread through occupation, ingesting contaminated drinking water and food that has been in contact with PFAS-coated packaging. Four PFAS (PFOA, PFOS, PFNA, and PFHxS) were detected in 98% of serum samples from humans over 12 in the US, indicating nearly universal exposure.^7^

PFOA and other PFAS are endocrine disruptors. The underlying biological mechanisms for PFOA exposure and cancers are an area of active research. However, a few studies have elucidated the mechanisms for PFOA and thyroid, female breast, prostate, and kidney and renal pelvis cancers and rhabdomyosarcoma in humans.^8-11^

Epidemiological studies that focus on potential connections between PFAS exposure and cancer are limited. A recent literature review found only 18 studies that included a quantitative estimate to measure PFAS and cancer.^12^ Several of the included studies are industry-sponsored. While currently available studies are informative, the evidence is not conclusive; but the most robust evidence supports the association between PFOA exposure and testicular and kidney cancer. Study designs limit the utility and suggest that population cohort studies would be powered sufficiently to contribute to our understanding of the connections between PFAS exposure and cancer. Importantly, PFAS serum measurement at the time of diagnosis does not accurately reflect PFAS levels connected with causation due to latency periods associated with cancer development and diagnosis.

Studies of health outcomes in 69 000 people exposed to PFOA from DuPont’s Washington Works plant in West Virginia concluded that PFOA exposure was “more probably than not” associated with testicular and kidney and renal pelvis cancers, ulcerative colitis, thyroid disease, hypercholesterolemia, and pregnancy-induced hypertension.^13^ Previous research indicates that PFOA exposure is also associated with female breast cancer, prostate, thyroid cancers, adverse reproductive outcomes, low birth weight, immune and endocrine disruption, and cardiovascular impacts.^14,15^ In addition, recent research indicates that a high blood level of PFAS is associated with an increased risk of severe disease after infection with the SARS-CoV-2 virus.^16-19^

A report from the NH Department of Health and Human Services (DHHS)^20^ attempted to evaluate the degree of exposure for individuals exposed to PFAS contaminated water in the Merrimack region. Blood samples were collected between 2016 and 2017 from 132 randomly selected addresses (resulting in 217 individuals sampled) who received their drinking water from the Merrimack Village District (MVD) and 219 individuals in southern NH with private wells with PFOA concentrations between 40 and 60 ppt. The geometric mean of PFOA blood samples was: 3.9 micrograms per liter (µg/L) for individuals using MVD public water and 4.4 µg/L for individuals using affected private wells. While prior work^1^ compared blood PFOA concentrations to national blood PFAS levels in 2013 to 2014, Figure 2 compares PFOA, PFOS, and PFHxS blood levels in Merrimack residents to mean US population blood levels between 2011 and 2017 since mean levels of these 3 PFAS have declined in recent years. Comparing only mean PFOA (not PFOS and PFHxS) blood concentrations to the mean US population levels in 2013 to 2014 likely underestimates the impact of PFAS exposure on Merrimack residents. The geometric PFOA means for blood samples from the 2 Merrimack cohorts are almost 3 times the national geometric mean of 1.56 µg/L between 2015 and 2016.^21^

**Figure 2.**
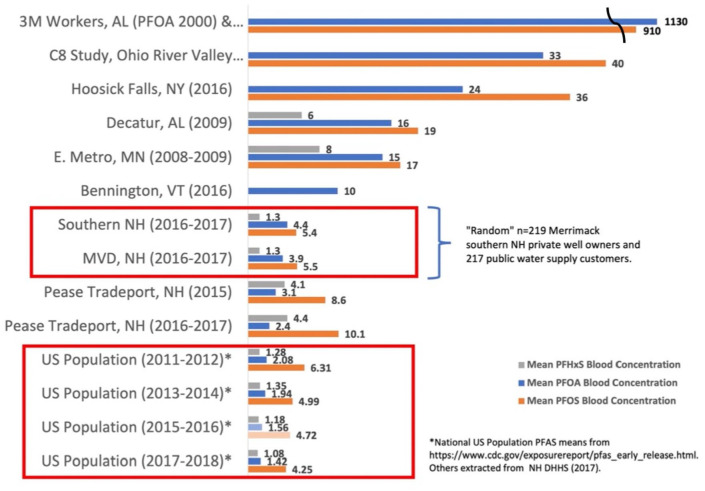
Mean PFHxS, PFOA, and PFOS blood concentrations. ^20,21^

Further analysis indicated that the geometric mean was higher in individuals who drank more than 8 cups of tap water per day and individuals who lived within 1.5 miles of the Saint Gobain plant.^20^ The DHHS concluded that mean PFOA serum levels in the small sample of Merrimack public water supply customers do not significantly differ from the US population.^20^ However, the mean PFOS serum concentration for Merrimack public water supply customers (5.5 µg/L) exceeds the US averages for 2013 to 2014 (5.0 µg/L).^20^ Our work is limited by the relatively small sample size and lack of data from individuals with private wells in the Merrimack Village District.^22^

The 2018 DHHS report indicated that the incidences of 24 cancer types in Merrimack over 10 years (2005-2014) are similar to incidence rates in general in NH. DHHS determined that Merrimack residents experience a 42% higher rate of kidney and renal pelvis cancers over 10 years (2009-2018) compared to the rest of NH.^23^ However, previous work did not compare Merrimack cancer incidence rates to national rates. In addition, cancer incidence rates in NH by town are not publicly available data to allow for comparison.

**Figure 3.**
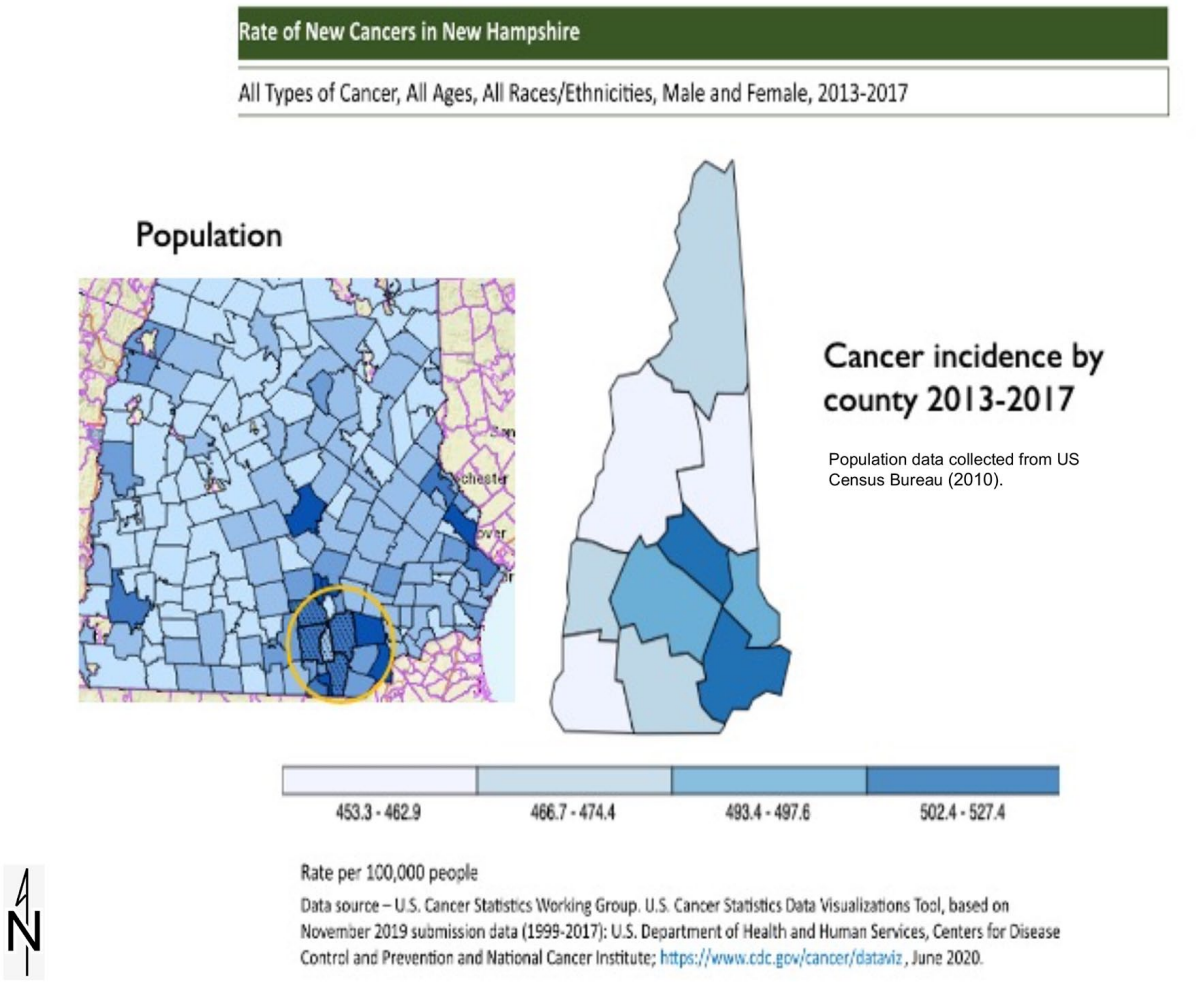
Population and cancer distribution in New Hampshire.

We hypothesize that Merrimack residents experience an increased risk of cancer compared to the general US population and similar New England communities without documented exposure to PFAS. Our analysis attempts to elucidate the risk of cancer in Merrimack, NH, by comparing to US rates and demographically similar communities with no documented widespread exposure to PFAS or other environmental contaminants. Additionally, Merrimack is located within a dense population center in NH (Figure 3). Statistically, this region contributes disproportionately to the state-wide cancer incidence rate. Thus, this analysis allows for a broader comparison and greater understanding of cancer risk Merrimack residents experience.

## Methods

Data for this ecological study included publicly available cancer incidence between January 2005 and December 2014,^1^ publicly available cancer incidence rates from the National Cancer Institute’s Surveillance, Epidemiology, and End Results (SEER) public database,^24^ and cancer incidences for comparator towns prepared by the Maine Cancer Registry, Data, Research and Vital Statistics, Maine CDC for ME communities. In addition, community-level cancer incidences for VT towns were obtained from the Vermont Health Surveillance System.^25^

### Exposure—PFAS contamination

Merrimack, NH, is the primary subject of the current analysis and is the community exposed to PFAS and other environmental contaminants. We selected 4 communities in Maine and Vermont with demographics (population, educational attainment, ethnicity, and median age) similar to Merrimack (Table 1) and no documented or suspected PFAS contamination (Table 2) as unexposed comparisons. The unexposed comparator towns included Sanford, Maine (ME), Auburn, ME, South Portland, ME, and Colchester, VT. In addition, Bennington, VT, was selected as an additional exposed community due to documented PFAS contamination for sensitivity analyses (described below in the analysis section) (Tables 1 and 2). Bennington, VT was chosen because of its demographic similarity (as outlined previously) to Merrimack, and both communities have documented PFAS contamination.

**Table 1.** Demographic characteristics of US, Merrimack, NH and comparator towns.

Demographic factor	South Portland, ME^a^	Auburn, ME^a^	Sanford, ME^a^	Colchester, VT^a^	Bennington, VT^a^	Merrimack, NH^a^	US Avg^a^
Population, 2010	25 002	23 055	20 798	17 067	15 764	25 494	308 745 538
Age							
Median Age, 2010	36	36.9	42.1	42.1	36.1	40.5	36.9
Persons under 18 years (%)	18	21	22	17	16	22	22
Persons 65 years and over (%)	17	18	17	13	22	15	17
Gender							
Female persons (%)	52	52	53	51	53	49	51
Race, ethnicity							
White alone (%)	90	91	93	93	94	92	76
Black or African American alone (%)	4	1	1	3	1	1	13
Asian alone (%)	2	1	3	2	1	2	6
Hispanic or Latino (%)	3	2	2	3	3	4	19
Economic factors							
Persons without health insurance, under age 65 years (%)	7	9	8	3	7	4	10
Median household income (in 2019 dollars), 2015-2019	$69 290	$49 719	$52 513	$71 090	$50 892	$107 232	$62 843
Persons in poverty (%)	9	11	14	11	15	4	11
Owner-occupied housing unit rate, 2015-2019 (%)	63	56	59	69	62	87	64
Living in same house 1 year ago, percent of persons age 1 year+, 2015-2019 (%)	87	83	83	81	87	91	86
Education							
High school graduate or higher, percent of persons age 25 years+, 2015-2019 (%)	96	91	92	95	90	97	88
Bachelor’s degree or higher, percent of persons age 25 years+, 2015-2019 (%)	46	29	19	43	32	47	32

a Demographic data obtained from https://www.census.gov/quickfacts/fact/table

**Table 2.** Summary of PFOA exposure in comparator drinking water.

Water district	PFOA in drinking water
Merrimack, NH^a^,*	140 parts per trillion (ppt)
South Portland, ME Water District^b^	2 ppt
Auburn, ME Water District^b^	Not detected (ND)
Sanford, ME Water District^b^	ND
Colchester, VT^c^	ND
Bennington, VT^c^	40 to 2880 ppt

a Merrimack data from: https://www4.des.state.nh.us/IISProxy/IISProxy.dll?ContentId=4649008

b ME data retrieved from: https://maine.maps.arcgis.com/apps/webappviewer/index.html?id=815b4093464c405daf7a17e43a1d9da7

c VT data retrieved from: https://www.healthvermont.gov/response/environmental/pfoa-drinking-water-2016

* Data listed for Merrimack Valley Water District well MW-4, which along with MW-5 was shut down in 2016 due to PFOA contamination.

### Outcome—Cancer incidence

We selected the same 24 cancer sites selected by DHHS^1^ and created an “all-cause cancer” incidence rate category, as others have done.^26,27^ We calculated the incidence for each cancer site and all-cause cancer in Merrimack, NH, over the 10 years from 2005 through 2014, except for lung and bronchus and prostate cancer. The incidences for lung and bronchus and prostate cancer were not available for Merrimack, NH for 2014; therefore, we calculated the incidences for those 3 cancer sites on 9 years of data, from 2005 to 2013. Gall bladder and Kaposi cancer are not included in the analysis due to the small sample size (less than 5 cases in the 10 years). Incidences were collected for each cancer type for the US general population and comparator communities and matched the timeframe available for cancer incidences in Merrimack, NH. Average incidence rates for each publicly available cancer site were time matched to the period used to calculate Merrimack average incidence rates.

Cancer incidence data for VT towns are limited to 7 cancer types and all-cause cancer, because those are the only data publicly available (bladder, colon, prostate, and female breast cancers, melanoma, lung and bronchus cancer, and non-Hodgkin Lymphoma).

In addition to analyzing the risk in Merrimack NH compared to each of the comparator communities separately, we combined data from each of the unexposed communities into a single “pooled” variable. We pooled the incidence data to increase study power to detect an effect and serve as a cross-reference consistency check of the increased or decreased risk pattern in comparator towns. A similar approach has been used by others, including Zahnd et al^26^ and Mastrantonio et al.^28^ The pooled variable includes all available data.

### Analysis

We ran unadjusted logistic regressions to approximate the risk ratio (RR) and 95% confidence intervals (CI) for each of the 24 cancer sites and all-cause cancer in Merrimack, NH compared to: (1) South Portland, ME, (2) Auburn, ME, (3) Sanford, ME, (4) Colchester, VT, (5) the pooled variable of unexposed communities, and (6) US average incidence.

We calculated precision estimates for any significant result to address the possibility of type 1 error. The precision estimate is equal to half of the width of the CI. Therefore, precision estimates less than 1.0 result from a narrow CI and increase our confidence in avoiding type 1 and type 2 errors.^29^

For our sensitivity analyses, we ran unadjusted logistic regressions to approximate the RR and 95% CI for each of the 7 available cancer sites (bladder, colon, prostate, and female breast cancers, melanoma, lung and bronchus cancer, and non-Hodgkin Lymphoma) and all-cause cancer in Merrimack, NH, compared to Bennington, VT, another community with documented PFAS contamination. We also ran unadjusted logistic regressions to approximate the RR and 95% CI for the 7 cancer types and all-cause cancer in Bennington, VT, compared to: (1) South Portland, ME, (2) Auburn, ME, (3) Sanford, ME, (4) Colchester, VT, (5) the pooled variable of unexposed communities, and (6) US average incidence.

All statistical analyses were performed using Stata/IC 16.1.^30^

## Results

**Figure 4.**
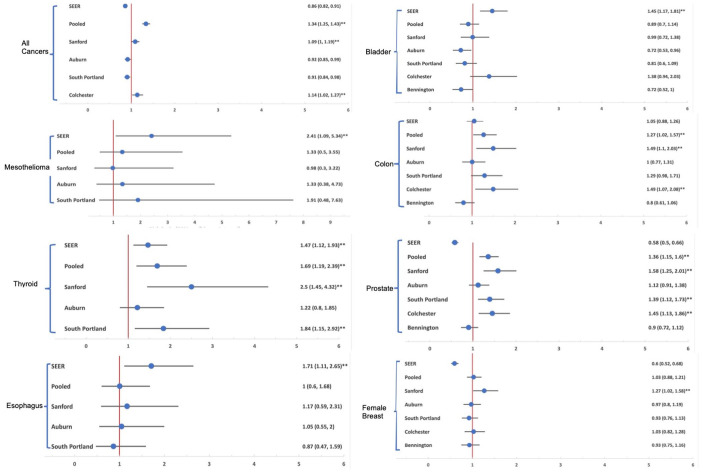
Risk ratios for Merrimack versus comparators.

Risk ratios and 95% CIs calculated for all-cause cancer types for unexposed comparators and exposed comparator towns are shown in Figure 4 and in Tables 3 and 4, respectively. Precision estimates (PE) are provided in Supplemental Table S-1.

**Table 3.** Risk ratios and 95% confidence intervals for cancer incidence in Merrimack, NH^a b^ versus unexposed communities, pooled unexposed communities, and the US average incidence.

	South Portland, ME^a,c^	Auburn, ME^a,c^	Sanford, ME^a,c^	Cochester, VT^a,d^	Pooled variable	US Avg incidence^a,e^
	RR	(95% CI)	RR	(95% CI)	RR	(95% CI)
All-cause cancer	**0.91 (0.84-0.98)**-	**0.92 (0.85-0.99)**-	**1.09 (>1-1.19)**+	**1.14 (1.02-1.27)**+	**1.34 (1.25-1.43)**+	**0.86 (0.82-0.91)-**
Bladder	0.81 (0.60**-**1.09)	**0.72 (0.53-0.96)**-	0.99 (0.72**-**1.38)	1.38 (0.94**-**2.03)	0.89 (0.70**-**1.14)	**1.45 (1.17-1.81)**+
Brain and other CNS	1.00 (0.55**-**1.82)	1.22 (0.64**-**2.33)	1.12 (0.59**-**2.14)	–	1.10 (0.67**-**1.82)	1.28 (0.84**-**1.95)
Cervix	0.37 (0.13**-**1.03)	0.49 (0.17**-**1.47)	2.04 (0.40**-**10.54)	–	0.55 (0.21**-**1.45)	**0.29 (0.12-0.69)**-
Colon	1.29 (0.98**-**1.71)	1.00 (0.77**-**1.31)	**1.49 (1.10-2.03)**+	**1.49 (1.07-2.08)**+	**1.27 (1.02-1.57)**+	1.05 (0.88**-**1.26)
Corpus and uterus	0.87 (0.61**-**1.26)	1.17 (0.78**-**1.74)	1.05 (0.70**-**1.56)	–	1.00 (0.74**-**1.38)	0.80 (0.61**-**1.04)
Esophagus	0.87 (0.47**-**1.59)	1.05 (0.55**-**2.00)	1.17 (0.59**-**2.31)	–	1.00 (0.60**-**1.68)	**1.71 (1.10-2.65)**+
Female breast	0.93 (0.76**-**1.13)	0.97 (0.80**-**1.19)	**1.27 (1.02-1.58)**+	1.03 (0.82**-**1.28)	1.03 (0.88**-**1.21)	**0.60 (0.52-0.68)**-
Hodgkin lymphoma	0.68 (0.22**-**2.15)	0.56 (0.18**-**1.70)	0.68 (0.21**-**2.23)	–	0.63 (0.24**-**1.68)	0.67 (0.28**-**1.61)
Kidney and renal pelvis	1.13 (0.75**-**1.17)	1.38 (0.89**-**2.13)	1.04 (0.69**-**1.58)	–	1.17 (0.84**-**1.63)	1.29 (0.98**-**1.69)
Larynx	0.61 (0.27**-**1.42)	0.57 (0.25**-**1.32)	0.82 (0.32**-**2.06)	–	0.65 (0.31**-**1.34)	1.12 (0.58**-**2.15)
Leukemia	1.00 (0.65**-**1.53)	**0.61 (0.41-0.89)**-	0.98 (0.63**-**1.52)	–	0.82 (0.58**-**1.15)	1.17 (0.86**-**1.57)
Liver and intrahepatic bile duct	**0.44 (0.22-0.87)**-	0.82 (0.37**-**1.80)	0.49 (0.24**-**>1.00)	–	0.54 (0.29 ->1.00)	0.58 (0.33**-**1.02)
Lung and bronchus	**0.60 (0.49-0.74)**-	**0.68 (0.54-0.85)-**	**0.65 (0.52-0.82)**-	1.05 (0.80**-**1.37)	**0.69 (0.58-0.83)**-	1.00 (0.85**-**1.18)
Melanoma	0.75 (0.53**-**1.04)	1.39 (0.93**-**2.08)	1.16 (0.79**-**1.71)	0.82 (0.57**-**1.20)	0.97 (0.73**-**1.29)	1.01 (0.79**-**1.30)
Mesothelioma	1.91 (0.48**-**7.63)	1.33 (0.38**-**4.73)	0.98 (0.30**-**3.22)	–	1.33 (0.50**-**3.55)	**2.41 (1.09-5.34)**+
Multle myeloma	0.70 (0.35**-**1.40)	0.62 (0.31**-**1.23)	1.14 (0.51**-**2.58)	–	0.76 (0.42**-**1.38)	0.82 (0.48**-**1.38)
Non-Hodgkin lymphoma	0.78 (0.53**-**1.14)	0.79 (0.54**-**1.17)	1.06 (0.69**-**1.63)	0.72 (0.48**-**1.09)	0.82 (0.60**-**1.13)	0.90 (0.68**-**1.19)
Oral cavity and pharynx	0.86 (0.52**-**1.44)	1.04 (0.60**-**1.79)	0.76 (0.46**-**1.28)	–	0.88 (0.57**-**1.34)	0.97 (0.67**-**1.40)
Ovary	0.77 (0.41**-**1.46)	0.69 (0.37**-**1.29)	0.99 (0.49**-**2.01)	–	0.79 (0.46**-**1.36)	**0.52 (0.33-0.84)**-
Pancreas	0.67 (0.42**-**1.06)	0.89 (0.54**-**1.48)	0.72 (0.44**-**1.18)	–	0.75 (0.50**-**1.12)	0.91 (0.64**-**1.13)
Prostate	**1.39 (1.12-1.73)**+	1.12 (0.91**-**1.38)	**1.58 (1.25-2.01)**+	**1.45 (1.13-1.86)**+	**1.36 (1.15-1.60)**+	**0.57 (0.50-0.66)**-
Stomach	0.73 (0.35**-**1.50)	0.64 (0.31**-**1.31)	0.89 (0.40**-**1.94)	–	0.74 (0.50**-**1.12)	0.70 (0.41**-**1.20)
Testes	0.64 (0.26**-**1.56)	1.02 (0.37**-**2.80)	3.27 (0.69**-**15.40)	–	1.01 (0.45**-**2.29)	0.51 (0.26**-**1.02)
Thyroid	**1.84 (1.15-2.92)**+	1.22 (0.80**-**1.85)	**2.50 (1.45-4.32)**+	–	**1.69 (1.19-2.39)**+	**1.47 (1.12-1.93)**+

Bold font and (+) indicates statistically significant higher rates while bold font and (-) indicates statistically significant lower rates.

a Calculated with average annual population between 2005-2014 (except for lung and prostate, 2005-2013). Annual population data obtained from: NH ( https://www.nh.gov/osi/data-center/population-estimates.htm), ME (https://www.maine.gov/dhhs/mecdc/public-health-systems/data-research/data/), VT (https://www.healthvermont.gov/health-statistics-vital-records/vital-records-population-data/vermont-population-estimates) and US (https://www.statista.com/statistics/183457/united-states–resident-population/).

b Merrimack, NH data obtained from DHHS 2018 .

c Maine cancer incidence data prepared by Maine Cancer Registry, Data, Research and Vital Statistics, Maine CDC 4/9/2021.

d Vermont data obtained from the state registry website (https://www.healthvermont.gov/stats/registries/cancer-registry).

^e^National incidence rates obtained by National Cancer Institute.^31^

**Table 4.** Risk ratios and 95% confidence intervals for cancer incidence in Merrimack, NH^a,b^ versus exposed community.

RR (95% CI)	Bennington, VT^a,c^
All-cause cancer	1.04 (0.95-1.14)
Bladder	0.72 (0.52**-**>1.00)
Brain and other CNS	–
Cervix	–
Colon	0.80 (0.61**-**1.06)
Corpus and uterus	–
Esophagus	–
Female breast	0.93 (0.75**-**1.16)
Hodgkin lymphoma	–
Kidney and renal pelvis	–
Larynx	–
Leukemia	–
Liver and intrahepatic bile duct	–
Lung and bronchus	**0.42 (0.33-0.52)**
Melanoma	0.82 (0.56**-**1.21)
Mesothelioma	–
Multiple myeloma	–
Non-Hodgkin lymphoma	**0.56 (0.38-0.83)**
Oral cavity and pharynx	–
Ovary	–
Pancreas	–
Prostate	0.90 (0.75**-**1.16)
Stomach	–
Testes	–
Thyroid	–

a Calculated with aaverage annual population between 2005-2014 (except for lung and prostate, 2005-2013). Annual population data obtained from: NH (https://www.nh.gov/osi/data-center/population-estimates.htm), ME (https://www.maine.gov/dhhs/mecdc/public-health-systems/data-research/data/), VT (https://www.healthvermont.gov/health-statistics-vital-records/vital-records-population-data/vermont-population-estimates) and US (https://www.statista.com/statistics/183457/united-states–resident-population/).

b Merrimack, NH data obtained from DHHS 2018.^1^

^c^Vermont data obtained from the state registry website (https://www.healthvermont.gov/stats/registries/cancer-registry).

### Merrimack, NH versus South Portland, ME

Residents of Merrimack, NH, have an 84% higher risk of thyroid cancer (RR = 1.84, 95% CI 1.15-2.92) and 39% higher risk of prostate cancer (RR = 1.39, 95% CI: 1.12-1.73) compared to residents of South Portland, ME. In contrast, residents of Merrimack have a 9% lower risk of all-cause cancer (RR = 0.91, 95% CI 0.84-0.98), 40% lower risk of lung and bronchus cancer (RR = 0.60, 95% CI 0.49-0.74), and 56% lower risk of liver and intrahepatic bile duct cancer (RR = 0.44, 95% CI 0.22-0.87) compared to residents of South Portland (Table 3). No association was found for Merrimack residents concerning the risk of any other specific types of cancer compared to residents of South Portland.

### Merrimack, NH versus Auburn, ME

Residents of Merrimack, NH, do not have a significantly higher risk of any type of cancer compared to residents of Auburn, ME. They do, however, have an 8% lower risk of all-cause cancer (RR = 0.92, 95% CI 0.85-0.99), 28% lower risk of bladder cancer (RR = 0.72, 95% CI 0.53-0.96), 39% lower risk of leukemia (RR = 0.61, 95% CI 0.41-0.89), and 32% lower risk of lung and bronchus cancer (RR = 0.68, 95% CI 0.54-0.85) compared to residents of Auburn (Table 3).

### Merrimack, NH versus Sanford, ME

Residents of Merrimack, NH have a 9% higher risk of all-cause cancer (RR = 1.09, 95% CI 1.00-1.19), 49% higher risk of thyroid cancer (RR = 2.5, 95% CI 1.45-4.32), 49% higher risk of colon cancer (RR = 1.49, 95% CI 1.10-2.03), 58% higher risk of prostate cancer (RR = 1.58, 95% CI 1.25-2.01), and 27% higher risk of female breast cancer (RR = 1.27, 95% CI 1.02-1.58) compared to residents of Sanford, ME (Table 3). However, the precision estimate for thyroid cancer exceeds 1.0, indicating that the result may be subject to type 1 error (Supplemental Table S-1). In contrast, residents of Merrimack have a 31% lower risk of lung and bronchus cancer (RR = 0.69, 95% CI 0.58-0.83) compared to residents of Sanford (Table 3). No association was found for Merrimack residents regarding the risk of any other specific types of cancer compared to residents of Sanford.

### Merrimack, NH versus Colchester, VT

The analysis comparing Merrimack, NH to Colchester, VT is limited to publicly available cancer incidence data (bladder, colon, prostate, and female breast cancers, melanoma, lung and bronchus cancer, and non-Hodgkin Lymphoma, and all-cause cancer). Residents of Merrimack have a 14% higher risk of all-cause cancer (RR = 1.14, 95% CI 1.02-1.27), 49% higher risk of colon cancer (RR = 1.49, 95% CI 1.07-2.08), and 45% higher risk of prostate cancer (RR = 1.45, 95% CI 1.13-1.86) compared to residents of Colchester (Table 3). No association was found for Merrimack residents regarding the risk of any of the other specific types of cancer available, compared to residents of Colchester.

### Merrimack, NH versus pooled data from unexposed communities

Residents of Merrimack, NH have a 34% higher risk of all-cause cancer (RR = 1.34, 95% CI 1.25-1.43), 69% higher risk of thyroid cancer (RR = 1.69, 95% CI 1.19-2.39), 27% higher risk of colon cancer (RR = 1.27, 95% CI 1.02-1.57), and 36% higher risk of prostate cancer (RR = 1.36, 95% CI 1.15-1.6) compared to the pooled risk of residents in 4 unexposed communities (Table 3). In contrast, residents of Merrimack have a 31% lower risk for lung and bronchus cancer (RR = 0.69, 95% CI 0.58-0.83) compared to the pooled risk of residents in 4 unexposed communities. No association was found for Merrimack residents regarding the risk of any other specific types of cancer compared to the pooled risk of residents in 4 unexposed communities.

### Merrimack, NH versus US average incidence

Residents of Merrimack, NH have a 141% higher risk of mesothelioma (RR = 2.41, 95% CI 1.09-5.34), 47% higher risk of thyroid cancer (RR = 1.47, 95% CI 1.12-1.93), 71% higher risk of esophageal cancer (RR = 1.71, 95% CI 1.1-2.65), and 45% higher risk of bladder cancer (RR = 1.45, 95% CI 1.17-1.81) compared to US national average risk (Table 3). In contrast, residents of Merrimack have 14% lower risk of all-cause cancer, (RR = 0.86, 95% CI 0.82-0.91), 43% lower risk of prostate cancer (RR = 0.57, 95% CI 0.5-0.66), 40% lower risk of female breast cancer (RR = 0.60, 95% CI 0.52-0.68), 48% lower risk of ovarian cancer (RR = 0.52, 95% CI 0.33-0.84), and 71% lower risk of cervical cancer (RR = 0.29, 95% CI 0.12-0.69) compared to US national average risk. However, the precision estimate for mesothelioma is greater than 1.0, indicating that the result may be subject to type 1 error (Supplemental Table S-1).

### Sensitivity analysis—Merrimack, NH versus Bennington, VT

We compared the risk of cancer in Merrimack with Bennington, VT, a community with similar documented PFAS exposure. Residents of Merrimack have a significantly lower risk of lung and bronchus cancer (RR = 0.42, 95% CI 0.33-0.52) and Non-Hodgkin Lymphoma (RR = 0.56, 95% CI 0.38-0.83) compared to residents of Bennington, but do not have a significantly different risk of any of the other types of cancer (Table 4).

### Sensitivity analysis—Bennington, VT versus comparators

We also compared the risk of cancer in Bennington, VT, with unexposed communities, pooled unexposed New England communities, and the US national average risk.

Residents of Bennington have a significantly higher risk of all-cause cancers (RR = 1.29, 95% CI 1.17-1.42), colon cancer (RR = 1.61, 95% CI 1.20-2.17), lung and bronchus cancer (RR = 1.44, 95% CI 1.19-1.75) and prostate cancer (RR = 1.54, 95% CI 1.22-1.96) compared to residents of South Portland, ME, but do not have a significantly different risk of any of the other types of cancer (Table 5).

**Table 5.** Risk ratios and 95% confidence intervals for cancer incidence in Bennington, VT^a c^ versus unexposed communities, pooled unexposed communties, and the US average incidence .

	South Portland, ME^a,b^	Auburn, ME^a,b^	Sanford, ME^a,b^	Cochester, VT^a,c^	Pooled variable^a^	US Avg incidence^a,d^
	RR (95% CI)	RR (95% CI)	RR (95% CI)	RR (95% CI)	RR (95% CI)	RR (95% CI)
All-cause cancer	**1.29 (1.17-1.42)**+	**1.29 (1.17-1.43)**+	**1.58 (1.42-1.76)**+	0.94 (0.85**-**1.04)	**1.41 (1.30-1.53)**+	**1.14 (1.06-1.22)**+
Bladder	1.12 (0.82**-**1.53)	0.99 (0.72**-**1.35)	1.37 (0.97**-**1.93)	1.38 (<1.00**-**1.91)	1.23 (0.94**-**1.61)	**2.00 (1.57-2.54)**+
Colon	**1.61 (1.20-2.17)**+	1.25 (0.94**-**1.67)	**1.87 (1.35-2.58)**+	1.25 (0.95**-**1.65)	**1.59 (1.25-2.01)**+	**1.31 (1.06-1.61)**+
Female breast	0.99 (0.80**-**1.24)	1.05 (0.83**-**1.31)	**1.36 (1.07-1.74)**+	1.07 (0.86**-**1.34)	1.10 (0.91**-**1.33)	**0.63 (0.53-0.75)**-
Lung and bronchus	**1.44 (1.19-1.75)**+	**1.63 (1.34-1.99)**+	**1.58 (1.29-1.93)**+	**2.41 (1.94-2.99)**+	**1.67 (1.43-1.96)**+	**2.41 (2.10-2.77)**+
Melanoma	0.91 (0.63**-**1.31)	**1.70 (1.10-2.60)**+	1.41 (0.93**-**2.15)	1.22 (0.83**-**1.79)	1.18 (0.86**-**1.63)	1.23 (0.92**-**1.65)
Non-Hodgkin lymphoma	1.39 (0.96**-**2.01)	1.42 (0.97**-**2.07)	**1.90 (1.25-2.89)**+	**1.79 (1.21-2.65)**+	**1.47 (1.08-2.00)**+	**1.61 (1.23-2.11)**+
Prostate	**1.54 (1.22-1.96)**+	1.24 (0.99**-**1.57)	**1.76 (1.36-2.27)**+	1.11 (0.89**-**1.38)	**1.50 (1.24-1.82)**+	**0.63 (0.54-0.75)**-

Bold font and (+) indicates statistically significant higher rates while bold font and (-) indicates statistically significant lower rates.

a Calculated with average annual population between 2005-2014 (except for lung and prostate, 2005-2013). Annual population data obtained from: NH (https://www.nh.gov/osi/data-center/population-estimates.htm), ME (https://www.maine.gov/dhhs/mecdc/public-health-systems/data-research/data/), VT (https://www.healthvermont.gov/health-statistics-vital-records/vital-records-population-data/vermont-population-estimates) and US (https://www.statista.com/statistics/183457/united-states–resident-population/).

b Maine data prepared by Maine Cancer Registry, Data, Research and Vital Statistics, Maine CDC 4/9/2021.

c Vermont data obtained from the state registry website (https://www.healthvermont.gov/stats/registries/cancer-registry).

d National incidence rates obtained by National Cancer Institute.^31^

Residents of Bennington have a significantly higher risk of all-cause cancers (RR = 1.29, 95% CI 1.17-1.43), lung and bronchus cancer (RR = 1.63, 95% CI 1.34-1.99) and melanoma (RR = 1.70, 95% CI 1.10-2.60) compared to residents of Auburn, ME, but do not have a significantly different risk of any of the other types of cancer (Table 5).

Residents of Bennington have a significantly higher risk of all-cause cancers (RR = 1.58, 95% CI 1.42-1.76), colon cancer (RR = 1.873, 95% CI 1.35-2.58), female breast cancer (RR = 1.36, 95% CI 1.07-1.74), lung and bronchus cancer (RR = 1.58, 95% CI 1.29-1.93), non-Hodgkin Lymphoma (RR = 1.90, 95% CI 1.25-2.89), and prostate cancer (RR = 1.76, 95% CI 1.36-2.27) compared to residents of Sanford, ME, but do not have a significantly different risk of any of the other types of cancer (Table 5).

Residents of Bennington have a significantly higher risk of lung and bronchus cancer (RR = 2.41, 95% CI 1.94-2.99) and non-Hodgkin Lymphoma (RR = 1.79, 95% CI 1.21-2.65) compared to residents of Colchester, VT, but do not have a significantly different risk of any of the other types of cancer (Table 5).

Residents of Bennington have a significantly higher risk of all-cause cancers (RR = 1.41, 95% CI 1.30-1.53), colon cancer (RR = 1.59, 95% CI 1.25-2.01), lung and bronchus cancer (RR = 1.67, 95% CI 1.43-1.96), non-Hodgkin Lymphoma (RR = 1.47, 95% CI 1.08-2.00), and prostate cancer (RR = 1.50, 95% CI 1.24-1.82) compared to the pooled risk of residents in 4 unexposed communities, but do not have a significantly different risk of any of the other types of cancer (Table 5).

Residents of Bennington have a significantly higher risk of all-cause cancers (RR = 1.14, 95% CI 1.06-1.22), bladder cancer (RR = 2.00, 95% CI 1.57-2.54), colon cancer (RR = 1.31, 95% CI 1.06-1.61), lung and bronchus cancer (RR = 2.41, 95% CI 2.10-2.77), and non-Hodgkin Lymphoma (RR = 1.61, 95% CI 1.23-2.11), and prostate cancer (RR = 1.50, 95% CI 1.24-1.82) compared to the US national average risk. Bennington residents have a significantly lower risk of female breast cancer (RR = 0.63, 95% CI 0.53-0.75) and prostate cancer (RR = 0.63, 95% CI 0.54-0.75), but do not have a significantly different risk of any of the other types of cancer (Table 5).

As previously noted, analyses for Vermont communities are limited to publicly available cancer incidence data (bladder, colon, prostate, and female breast cancers, melanoma, lung and bronchus cancer, and non-Hodgkin Lymphoma, and all-cause cancer).

## Discussion

We analyzed the risk of 24 types of cancer and all-cause cancer in Merrimack, NH, after community concern due to documented exposure to PFAS in air and drinking water from an industrial source.

Results indicate that Merrimack residents have a 47% higher risk of thyroid cancer compared to the general US population (RR = 1.47, 95% CI 1.12-1.93) and a 69% higher risk than the pooled risk of residents in 4 unexposed towns (RR = 1.69 95% CI 1.19-2.39). Merrimack residents also have an 84% increased risk for thyroid cancer compared to South Portland and a 150% higher risk for thyroid cancer when compared to Sanford, ME. These results suggest a unique factor contributing to thyroid cancer risk in Merrimack, which may be contributing to causation, such as PFAS exposure. While we could not identify documented PFAS contamination of the water supply in Auburn, ME, it was identified as a priority PFAS investigation community based on knowledge of sludge, septic tank sewage, and industrial waste spreading practices.^32^ Residents of Auburn, ME, receive drinking water from public supplies but also private wells. As of May of 2021, a similar investigation in Fairfield, ME, uncovered 63 private wells with PFAS concentrations above the USEPA advisories relating to sludge spreading practices.^33^ Thus, it is plausible that Auburn residents have unrecognized PFAS exposure that could contribute to cancer risk similar to Merrimack residents.

PFOA exposure is associated with incident nonmalignant thyroid disease^34-40^ and disruptions in thyroid hormone levels due to prenatal exposure.^41^ In addition, one study found a possible trend between PFOA exposure and thyroid cancer,^42^ and another found a dose-related relationship between PFOA exposure and thyroid cancer^43^ however, results of both studies have limited applicability due to study design.^12^

**Figure 5.**
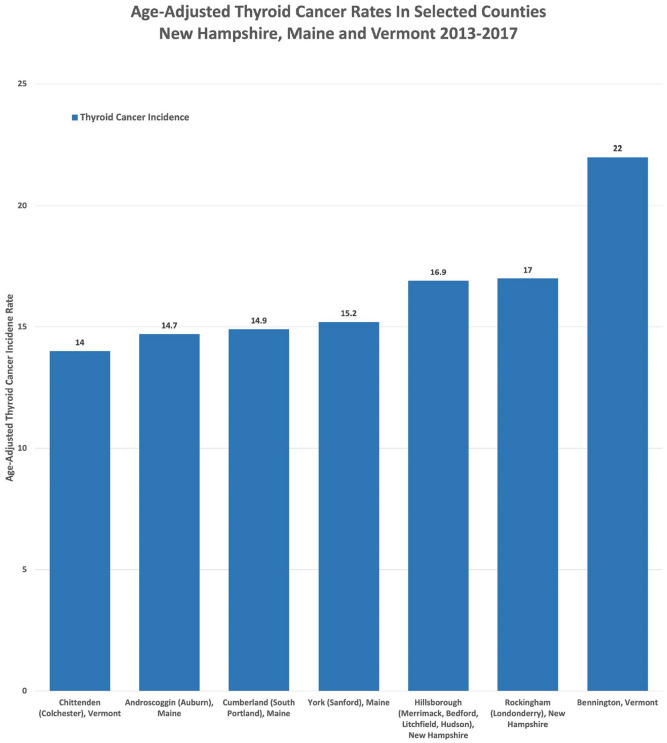
Age-adjusted thyroid cancer for NH, VT, and ME counties.^58^

As shown in Figure 5, thyroid cancer rates in Bennington County, VT (Bennington) are higher than the comparator town counties (Rockingham and Hillsborough, NH and Chittenden, VT) in ME and VT, where there is no evidence of PFOA contamination in the water supply. The county rates of thyroid cancer in Bennington, VT, are higher than the ME towns and similar to Hillsborough and Rockingham counties, NH, where there are documented cases of widespread PFAS in the drinking water supply.

Merrimack residents have a 45% increased risk for bladder cancer, 71% increased risk for esophageal cancer, and 141% increased risk for mesothelioma than the pooled risk for the 4 New England towns. Thus, while PFAS may contribute to higher than US average risk for bladder and esophageal cancers and mesothelioma, other regional factors contributing to the cancers, such as arsenic exposure, cannot be ruled out.

Previous studies that have examined potential associations between PFOA and bladder cancer report conflicting results. Several studies have found modest but not statistically significant connections between PFOA exposure and bladder cancer.^28^ One study conducted in a Danish cohort did not find an association between PFOA plasma levels and elevated risk of developing bladder cancer.^44^ However, a study of PFOA employees in Decatur, Alabama, identified a higher rate of bladder cancer death in fluorochemical workers with high PFOA exposure jobs.^45^

**Figure 6.**
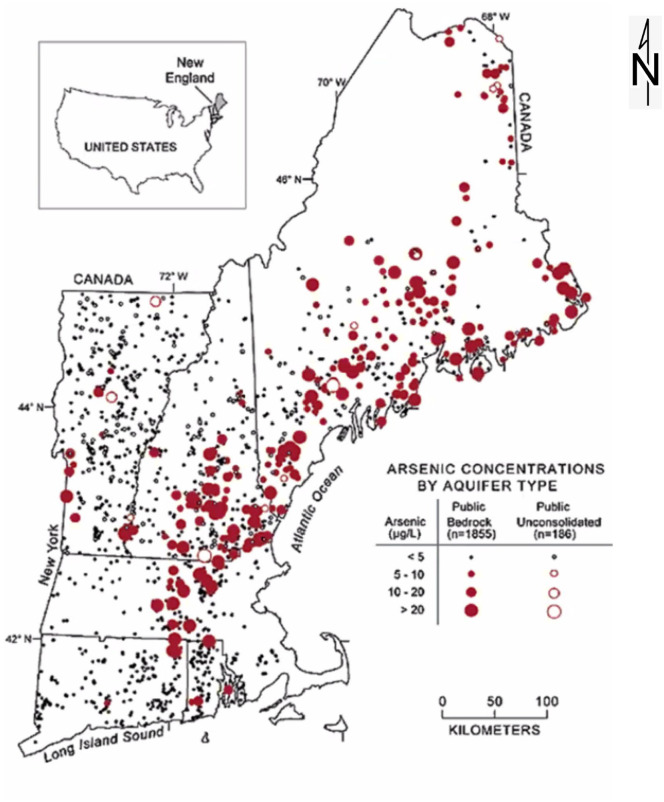
Arsenic concentrations in drinking water in New England.^48^

Merrimack and Maine residents could be exposed to arsenic in their drinking water, which may increase their risk of developing bladder cancer from private wells^46^ and municipal sources.^47^ Arsenic levels above 10 parts per billion in drinking water wells are widespread in New Hampshire and Maine, as shown in Figure 6.^48^ In New Hampshire, the maximum contaminant level (MCL) for drinking water was lowered to 0.05 µg/L in 2019^49^ to reduce the rate of bladder and lung cancers.

Mid-Ohio Valley residents exposed to PFOA in drinking water due to industrial emissions did not have significantly elevated esophageal cancer hazard ratios with PFOA exposure.^42^ We did not locate other studies examining the potential connections between PFOA exposure and esophageal cancer; however, our results show that Merrimack residents experience a 71% higher risk for esophageal cancer, so further study is warranted.

The 141% increased risk for mesothelioma but similar to the pooled risk, may suggest a unique exposure to Merrimack residents, but a regional factor, such as asbestos exposure, cannot be ruled out. A previous study of DuPont workers from 8 states found a significant correlation between elevated PFOA serum levels and standard mortality rates (SMR) for mesothelioma.^50^ We did not find other studies examining PFAS exposure and mesothelioma. Since there are only 6 mesothelioma cases in Merrimack, investigations should examine what factors contributed to the cancers. In addition, DuPont workers in the study may have been exposed to glass fiber materials in addition to PFOA^50^.

Studies have suggested a potential link between fiberglass exposure and mesothelioma in boat builders.^51^ Since Saint Gobain appears to have continued ChemFab’s production of fiberglass coated fabrics, and boatbuilding is prominent in New England towns, we suggest that case-control studies may be informative to assess potential causes for mesothelioma in these industries.

Merrimack residents also experience a 14% higher risk of all-cause cancers, 27% higher risk for colon cancer, and 36% higher risk for prostate cancer when compared to pooled data from 4 comparator New England towns without documented exposure to PFAS but a 43% lower risk for prostate cancer than the US average. Merrimack residents have a higher risk for each of these cancers in most cases compared to each unexposed New England town, except Auburn, ME. As previously suggested, there may be unrecognized PFAS exposure in Auburn, ME, contributing to the cancer risk causing a subdued effect on the pooled variable and when compared separately with Merrimack risk.

Bennington, VT has documented PFAS contamination, specifically from the St. Gobains factory. If PFAS contamination does indeed increase the risk of all-cause and some specific cancers, we would expect to see similarly increased risk profiles for all-cause cancer and specific cancer sites in Bennington and Merrimack, as compared to unexposed communities, the pooled variable, and the US national average risk. The results of our sensitivity analysis demonstrate that Bennington and Merrimack do have similar risk profiles. Residents of Bennington, VT have an increased risk of all-cause cancers (41%), 59% increase for colon cancer, and 50% increased risk for prostate cancer compared to pooled data from 4 comparator New England towns without documented exposure to PFAS. Bennington residents also have a 14% increased risk for all-cause cancers, 100% increased risk for bladder cancer, 31% increased risk for colon cancer when compared with the US average but 37% reduced risk for prostate cancer. In addition, DHHS recently determined that between 2008 and 2019 Merrimack residents experience elevated risk for non-Hodgkin Lymphoma when compared to the rest of the state.^23^ The similarity of results found in Merrimack and Bennington provide additional support to validity of the pattern of results found in this study.

An in-vitro study found that PFOA could induce colorectal cancer^52^; however, in vivo studies have not found an association between colorectal cancer and PFOA exposure^42,43^ or possibly a lower risk association from exposure to PFOA.^53^ In the latter study, serum PFOA concentrations were assessed at diagnosis, raising concerns about latency, and the paper provides insufficient information to evaluate reverse causation.^12^ Nevertheless, our results indicate that Merrimack residents experience a significantly higher risk of colon cancer when compared to pooled data from New England towns that have no documented exposure to PFAS in drinking water supplies. Therefore, further study regarding the elevated colon cancer risk in Merrimack residents is warranted.

Prostate cancer is, by far, the highest reported incident cancer in workers exposed to PFOA at the Cottage Grove and Saint Paul, Minnesota 3M factories.^54^ Another study found an inconsistent dose-related relationship between standard mortality rates (SMRs) for prostate cancer and cumulative PFOA exposure among workers at the Cottage Grove, Minnesota 3M factory.^55^ Another ecological mortality study did not find a relationship between mortality and prostate cancer.^28^ However, since prostate cancer screening and treatments have improved, mortality studies are not likely to help identify PFAS exposure connections with prostate cancer.

The sensitivity analysis results indicate the risk for female breast and prostate cancers for Merrimack residents is similar to Bennington, VT, a community with extensive documented PFOA in the drinking water supply. Before moving to Merrimack, air emissions from Saint Gobain’s operations contaminated water supplies in Bennington, VT.

A small occupational cohort study with occupational exposure to PFOA found an increased risk for liver cancer and malignant neoplasms of lymphatic and hematopoietic tissue associated with high internal doses of PFOA.^56^ In our study, Merrimack residents have a 56% reduced risk for liver and intrahepatic bile duct cancer compared with South Portland, ME; however, Merrimack residents do not have significantly different risk compared to pooled risk, US average risk, or other New England towns.

### Strengths and limitations

To our knowledge, our approach is novel because we compare cancer incidences in a community exposed to PFAS in air and drinking water supplies to the US general population and demographically similar towns with and without documented PFAS contamination drinking water supplies. While the approach is unique, the analysis is responsive to concerns raised by community members, especially where this region may contribute disproportionately to the state-wide cancer incidence rate. Our results recognize that community concerns may be valid and not fully addressed only by comparison with state-wide incidence rates. Our approach provides a roadmap for further study. It suggests a technique that more adequately addresses the concerns of communities facing environmental exposures that could provide a roadmap for proactive measures to prevent cancer and chronic illness.

As previously indicated, the southern NH area with documented PFAS in the water supply encompasses a minimum of 5 towns and is in a densely populated area of the state (Figure 3) and comprises approximately 33% of the state population. Therefore, if exposure to PFAS increases cancer risk, we would expect cancer rates to be elevated in Merrimack and the state of NH. Consequently, it is reasonable to expect that we would see no statistically significant differences between the community of Merrimack and the state of NH. Therefore, comparing the risk of cancer in Merrimack to the general US population and similar communities without PFAS exposure is more accurate.

The findings in this report are subject to at least 5 limitations. First, we cannot compare the risk of cancer in Merrimack to the risk of cancer in the state of NH or for other periods because the data are not publicly available. A possible critique of our results is that if this comparison were possible, the risk in Merrimack would not be significantly different from the state-wide risk. However, the state of NH experiences relatively high rates of pediatric, bladder, female breast, esophageal and other cancers, compared to the general US population and other states.^57^ We do not believe this limits interpretation of our results. In addition, more granular demographic information relating to the cancer cases in Merrimack, NH, is not publicly available.

Similarly, town-level cancer incidence for all 24 cancers is not publicly available for VT comparator towns. Therefore, our analysis included comparisons for incidences of 7 publicly available cancers, including bladder, colon, female breast, and prostate cancers, melanoma, lung and bronchus cancer, and non-Hodgkin Lymphoma. More extensive analysis for all 24 cancer types comparing incident risk between Merrimack and Bennington, VT may be instructive, especially since widespread documented PFOA in the water supply and blood levels suggest residents in Merrimack and Bennington have similar exposure to PFAS. A similar limitation in obtaining cancer incidence data for VT was reported by Zahnd et al.^26^

Second, delays in cancer reporting can result in underestimating certain cancers in the case of prostate and lung and bronchus cancer due to completeness limitations reported in the original study where the Merrimack, NH data were derived.^1^

Thirdly, some unrecognized PFAS or other environmental exposure may contribute to cancer incidence in our “unexposed towns” that we are unaware of, resulting in underestimating the risk of cancer incidence in Merrimack residents. The previous discussion regarding potential PFAS exposure in Auburn, ME, exemplifies how this limitation may impact our results.

Fourthly, our analysis has many comparisons, and we cannot rule out the risk of type 1 error. However, the consistency of results and results of the sensitivity analysis point to the validity of our findings. For each of the national and state comparisons, it is expected that, on average, there could be one false positive, at the *P* < .05 significance level, among a comparison of the 24 cancer types. Except for mesothelioma risk for Merrimack versus national incidence and risk of thyroid in Merrimack compared to Sanford, ME, our precision estimates do not suggest Type I error, as shown in Supplemental Table S-1. The precision estimate for mesothelioma is low but not unexpected because it is relatively rare, and the confidence interval is relatively wide. Thyroid risk is significantly higher for Merrimack residents than the US population, pooled towns, and South Portland, Maine; therefore, further study of thyroid cancers in the Merrimack area is warranted.

Finally, since we use population-level data in our ecological study, comorbid, behavioral, or other risk factors contributing to cancer risk are unknown. Nevertheless, ecological studies help develop hypotheses and confirm the need for individual-level data. Therefore, further case-control studies should examine the association between exposure to PFAS in the Merrimack, NH community and the risk of mesothelioma and esophageal, thyroid, bladder, colon, and prostate cancers.

## Conclusions

A previous cancer incidence analysis did not find elevated risk for Merrimack residents compared with state-wide incidence rates. We argue that state-wide incidence rates were not the best comparator due to NH’s unique context having multiple PFAS-impacted towns, which may significantly influence state-wide incidence rates. These towns in the southern NH region contribute a large proportion of the population of the state. Additionally, state averages for some cancers are the highest in the nation (bladder, female breast, esophageal).^31^

Our ecological study separately compared Merrimack cancer incidences between 2005 and 2014 to national cancer incidence rates, pooled incidence rates for 4 New England towns and separately with 3 municipalities in ME for 24 cancer types and all-cause cancers and 2 towns in VT for 7 cancer types and all-cause cancers.

Merrimack residents experience significantly higher rates of mesothelioma and esophageal, thyroid, and bladder cancers between 2005 and 2014 compared to US incidence rates.

The present study suggests that Merrimack citizens experience significantly higher risks for developing environmentally triggered cancers (ie, thyroid, colon, and prostate) and all-cause cancers than pooled cancer incidence for New England towns with no documented PFAS contamination in the water supply. In addition, our results also suggest that Merrimack residents may be at significantly higher risk for female breast cancer than residents of Sanford, ME.

This study also suggests that female breast and prostate cancer rates may be similar to Bennington, VT. Additionally, thyroid cancer rates at the town level may be similar to Bennington, VT, based on county-level data. Saint Gobain moved from Bennington, VT in 2002, where industrial airborne emissions caused widespread PFOA-drinking water contamination and documented exposure in Bennington, VT residents. Our results suggest that further study is warranted comparing cancer rates in Merrimack residents to other similarly PFOA-exposed towns, like Bennington, VT.

To summarize, further research relating to cancer risk in Merrimack is suggested by this study. Interestingly, though limited by public data availability, our study indicates that Merrimack residents experience a similar risk for prostate cancer and possibly thyroid cancer as Bennington, VT; both towns have documented PFAS contamination of drinking water supplies. Unfortunately, we could not obtain town-level cancer incidence data for many of the 24 types we analyzed for VT towns to conduct further analysis; however, our results suggest further study is warranted.

Finally, our work suggests further investigation, including case-control and cohort studies, is warranted to identify causative exposures that may be contributing to cancer and chronic disease to inform policy measures and protect public health. A proactive approach is critical to understanding the risk associated with PFAS and other environmental exposures and developing strategies to reduce cancer risk.

## Supplemental Material

sj-pdf-1-ehi-10.1177_11786302221076707 – Supplemental material for Risk of Cancer in a Community Exposed to Per- and Poly-Fluoroalkyl Substances Click here for additional data file. Supplemental material, sj-pdf-1-ehi-10.1177_11786302221076707 for Risk of Cancer in a Community Exposed to Per- and Poly-Fluoroalkyl Substances by Mindi F Messmer, Jeffrey Salloway, Nawar Shara, Ben Locwin, Megan W Harvey and Nora Traviss in Environmental Health Insights
