# Supplementary material for: Risk of Cancer in a Community Exposed to Per- and Poly-Fluoroalkyl Substances
Source: Environ Health Insights. 2022 Feb 11;16:11786302221076707. doi: 10.1177/11786302221076707 (PMC8842173; doi:10.1177/11786302221076707)
Supplement: sj-pdf-1-ehi-10.1177_11786302221076707 – Supplemental material for Risk of Cancer in a Community Exposed to Per- and Poly-Fluoroalkyl Substances [file sj-pdf-1-ehi-10.1177_11786302221076707.pdf]

Title: Risk of Cancer in a Community Exposed to Per- and Poly-Fluoroalkyl Substances

Authors: *Mindi F. Messmer*[1,2]; Jeffrey Salloway, PhD[3]; Nawar Shara, PhD[4,5]; Ben Locwin, PhD[6]; Megan W. Harvey, PhD[7]; Nora Traviss, PhD[2,8]

Author Affiliations: 1- VistaNova Consulting; 2- NH Science and Public Health; 3- Department of Health Management and Policy, University of New Hampshire; 4- Georgetown-Howard Universities Center for Clinical and Translational Science, Washington, DC; 5- MedStar Health Research Institute, Washington, DC 6- Private Consultant; 7- Springfield College, School of Health Sciences; 8- Environmental Studies, Keene State College

Supplemental Information

Table S-1. Precision Estimates for Confidence Intervals

Cancer Type/Site	South Portland, ME[a]	Auburn, ME[a]	Sanford, ME[a]	Colchester, VT[a]	Pooled Variable[a]	US Avg Incidence[a]
Mesothelioma	—	—	—	—	—	2.14
Esophagus	—	—	—	—	—	**0.78**
Thyroid	**0.88**	—	1.43	—	**0.6**	**0.41**
Bladder	—	**0.22**	—	—	—	**0.32**
Colon	—	—	**0.47**	**0.5**	**0.28**	—
Female Breast	—	—	**0.28**	—	—	—
Prostate	**0.31**	—	**0.38**	**0.36**	**0.23**	—
Testes	—	—	—	—	—	—
Kidney and Renal Pelvis	—	—	—	—	—	—
All Cancers	—	—	**0.095**	**0.13**	**0.09**	—

[a]Precision estimates calculated based on methods in Benjamini & Yekutieli, 2005.
